# The glycoprotein, non-virion protein, and polymerase of viral hemorrhagic septicemia virus are not determinants of host-specific virulence in rainbow trout

**DOI:** 10.1186/s12985-019-1139-3

**Published:** 2019-03-07

**Authors:** Shamila Yusuff, Gael Kurath, Min Sun Kim, Tarin M. Tesfaye, Jie Li, Douglas G. McKenney, Vikram N. Vakharia

**Affiliations:** 1 Institute of Marine & Environmental Technology, University of Maryland Baltimore County, 701 E. Pratt Street, Baltimore, MD 21202 USA; 2 U.S. Geological Survey, Western Fisheries Research Center, 6505 NE 65th Street, Seattle, WA 98115 USA; 3 GeneDX 207 Perry Parkway, Gaithersburg, MD 20877 USA; 4 Department of Integrative Bio-Industrial Engineering, Sejong University, Seoul, Republic of South Korea

**Keywords:** VHSV, Virulence determinant, Fish, Rhabdovirus, Glycoprotein, Non-virion protein, Polymerase protein

## Abstract

**Background:**

Viral hemorrhagic septicemia virus (VHSV), a fish rhabdovirus belonging to the *Novirhabdovirus* genus, causes severe disease and mortality in many marine and freshwater fish species worldwide. VHSV isolates are classified into four genotypes and each group is endemic to specific geographic regions in the north Atlantic and Pacific Oceans. Most viruses in the European VHSV genotype Ia are highly virulent for rainbow trout (*Oncorhynchus mykiss*), whereas, VHSV genotype IVb viruses from the Great Lakes region in the United States, which caused high mortality in wild freshwater fish species, are avirulent for trout. This study describes molecular characterization and construction of an infectious clone of the virulent VHSV-Ia strain DK-3592B from Denmark, and application of the clone in reverse genetics to investigate the role of selected VHSV protein(s) in host-specific virulence in rainbow trout (referred to as trout-virulence).

**Methods:**

Overlapping cDNA fragments of the DK-3592B genome were cloned after RT-PCR amplification, and their DNA sequenced by the di-deoxy chain termination method. A full-length cDNA copy (pVHSVdk) of the DK-3592B strain genome was constructed by assembling six overlapping cDNA fragments by using natural or artificially created unique restriction sites in the overlapping regions of the clones. Using an existing clone of the trout-avirulent VHSV-IVb strain MI03 (pVHSVmi), eight chimeric VHSV clones were constructed in which the coding region(s) of the glycoprotein (G), non-virion protein (NV), G and NV, or G, NV and L (polymerase) genes together, were exchanged between the two clones. Ten recombinant VHSVs (rVHSVs) were generated, including two parental rVHSVs, by transfecting fish cells with ten individual full-length plasmid constructs along with supporting plasmids using the established protocol. Recovered rVHSVs were characterized for viability and growth in vitro and used to challenge groups of juvenile rainbow trout by intraperitoneal injection.

**Results:**

Complete sequence of the VHSV DK-3592B genome was determined from the cloned cDNA and deposited in GenBank under the accession no. KC778774. The trout-virulent DK-3592B genome (genotype Ia) is 11,159 nt in length and differs from the trout-avirulent MI03 genome (pVHSVmi) by 13% at the nucleotide level. When the rVHSVs were assessed for the trout-virulence phenotype in vivo, the parental rVHSVdk and rVHSVmi were virulent and avirulent, respectively, as expected. Four chimeric rVHSVdk viruses with the substitutions of the G, NV, G and NV, or G, NV and L genes from the avirulent pVHSVmi constructs were still highly virulent (100% mortality), while the reciprocal four chimeric rVHSVmi viruses with genes from pVHSVdk remained avirulent (0–10% mortality).

**Conclusions:**

When chimeric rVHSVs, containing all the G, NV, and L gene substitutions, were tested in vivo, they did not exhibit any change in trout-virulence relative to the background clones. These results demonstrate that the G, NV and L genes of VHSV are not, by themselves or in combination, major determinants of host-specific virulence in trout.

## Background

Viral hemorrhagic septicemia virus (VHSV) is the causal agent of a serious disease in many marine and freshwater fish species worldwide [1–3]. The virus produces severe hemorrhages in the skin, muscles, eyes, kidney and liver, with mortality rates as high as 90% [3]. VHSV is an enveloped, non-segmented, negative-sense RNA virus in the *Novirhabdovirus* genus of the *Rhabdoviridae* family, species *Piscine novirhabdovirus* [4, 5]. Its genome is composed of approximately 11-kb of single-stranded, negative-sense RNA, which contains six genes that are positioned along the genome in the 3′ to 5′ order: 3′-N-P-M-G-NV-L-5′, nucleocapsid protein (N), polymerase-associated phosphoprotein (P), matrix protein (M), surface glycoprotein (G), a unique non-virion protein (NV) and viral polymerase (L) [6, 7]. The functions ascribed to novirhabdovirus structural proteins are largely based on the studies conducted on the mammalian rhabdoviruses, namely, rabies and vesicular stomatitis virus [4, 8]. N is the major structural protein which tightly binds the RNA genome. L protein is responsible for the virion-associated RNA transcription and genome replication activity. P protein is responsible for binding L protein to the N protein-RNA template. N, P and L proteins together form the functional viral polymerase complex. The M protein condenses the nucleocapsid into a tightly coiled nucleocapsid-M protein complex, which gives the virion bullet-like shape. The G protein is the major surface antigen on viral particles and it interacts with cell receptors to facilitate cell entry. For VHVS and the related fish novirhabdovirus, infectious hematopoietic necrosis virus (IHNV), it has been shown that the G protein interacts with the host cell receptors and induces neutralizing antibodies in infected fish [9, 10]. Distinct from mammalian rhabdovirus genomes, fish novirhabdovirus genomes contain an additional gene found between the G and L cistrons that codes for the NV protein. This NV protein is required for efficient replication and pathogenicity in fish [11–13], and it also suppresses apoptosis and innate immune responses [14–16].

Viral hemorrhagic septicemia (VHS) disease, first described in freshwater-reared rainbow trout (*Oncorhynchus mykiss*) in Europe in 1938, has continued to plague the European trout farming industry since the 1950s [1, 2, 17]. In the late 1980s, VHSV was isolated for the first time in western North America from adult Coho salmon and Pacific cod showing hemorrhagic lesions [18]. In 2005, a new genotype of VHSV emerged in the Great Lakes region of the United States and Canada, which caused massive mortality in many freshwater species [19–23].

VHSV isolates are classified into four genotypes based on phylogenetic analyses of the G and N sequences [24, 25]. Genotype I is divided into six sub-genotypes (Ia to If), with the Ia strain being virulent in rainbow trout and responsible for most outbreaks in European freshwater trout farms [24–27]. Genotype II and III viruses are endemic in wild marine fish in Europe [24, 25, 27–29]. Genotype IV is divided into three sub-genotypes: IVa (Asia and North American Pacific Coast), IVb (Great Lakes of North America), and IVc (North American Atlantic Coast) [19, 20, 22, 30–32]. The virulence of VHSV isolates is host-specific and varies generally with the viral genotypes and sub-genotypes. European genotype Ia and Ic isolates are virulent for rainbow trout, whereas VHSV marine isolates of genotype Ib, II and III are nearly all avirulent or low virulence in rainbow trout [1, 27, 33]. On the other hand, genotype IV isolates are virulent in several wild marine and freshwater fish species, but they are not virulent in rainbow trout [18, 30, 34, 35].

To identify the molecular basis of VHSV virulence in rainbow trout, several researchers sequenced the VHSV genomes of virulent and avirulent isolates from Europe, and compared the amino acid sequences of all six VHSV proteins [36, 37]. Only 14 amino acid substitutions were identified among the selected virulent and avirulent marine isolates, of which five occurred in the N protein, three each in the P and L proteins, two in the G protein, and one in the NV protein [36, 37]. Other researchers have suggested specific amino acids in the N gene as candidate determinants for virulence in rainbow trout, based on reactivity patterns of N-specific monoclonal antibodies and comparisons of genomes sequences for VHSV isolates in genotypes Ib and III [38, 39]. In addition, some insight into the genetic basis of novirhabdovirus virulence in rainbow trout has been generated from reverse genetics studies based on an infectious clone of a different fish novirhabdovirus species, infectious hematopoietic necrosis virus (IHNV), in which chimeric recombinant viruses containing genes from other fish rhabdoviruses, including VHSV, have been tested for virulence in vivo [40–43]. However to date, the individual VHSV viral protein(s) responsible for host-specific virulence in rainbow trout (referred hereafter as trout-virulence) have not been identified.

In a previous study, we generated an infectious clone (referred to here as pVHSVmi) of the trout-avirulent Great Lakes VHSV strain MI03 (genotype IVb) and demonstrated that the recovered recombinant VHSV was highly virulent in one of its natural host species, yellow perch (*Perca flavescens*) [13]. Here, we prepared an infectious clone of the trout-virulent European genotype Ia strain DK-3592B (pVHSVdk) to provide the contrasting phenotype for investigating the genetic basis of trout-virulence. Eight chimeric VHSV clones were constructed in which the coding region(s) of glycoprotein (G), non-virion protein (NV), G and NV, and G, NV and polymerase (L) genes were exchanged between the two clones. Using the cRNA-based reverse genetics system for VHSV, ten recombinant VHSVs (rVHSVs) were generated, including two parental VHSVs, from the full-length plasmids [13]. In this report, we describe the characteristics of these recovered viruses in vitro and ascertain the role of VHSV viral protein(s) G, NV, and L in trout-virulence by conducting experiments in rainbow trout.

## Materials and methods

### Viruses and cells

The North American MI03 strain of VHSV (genotype IVb) [19] was originally from Dr. M. Faisal at Michigan State University, and the European DK-3592B reference strain of VHSV (genotype Ia) [33] was provided by Drs. K. Einer-Jensen and N. Lorenzen of the Danish Technical University. These parental virus strains and all recombinant VHSVs generated here were propagated in the *Epithelioma papulosum cyprini* (EPC) fish cell line [44] at 14 °C. The cells were grown at 25 °C in minimal essential medium (MEM) supplemented with 10% fetal bovine serum (FBS) and 2 mM L-glutamine. For preparation of wild type or recombinant virus stocks, confluent EPC cells grown at 25 °C were infected with virus at 14 °C at a multiplicity of infection (MOI) of 0.01 in MEM with 2% FBS. After 1 h of adsorption at 14 °C, the inoculum was removed, and the cells were incubated at 14 °C until extensive cytopathic effect (CPE) was observed. The supernatant was collected 4–5 days post-infection, clarified and stored at − 80 °C for further use.

### Construction of a full-length cDNA clone of the European DK-3592B strain of VHSV

**Fig. 1:**
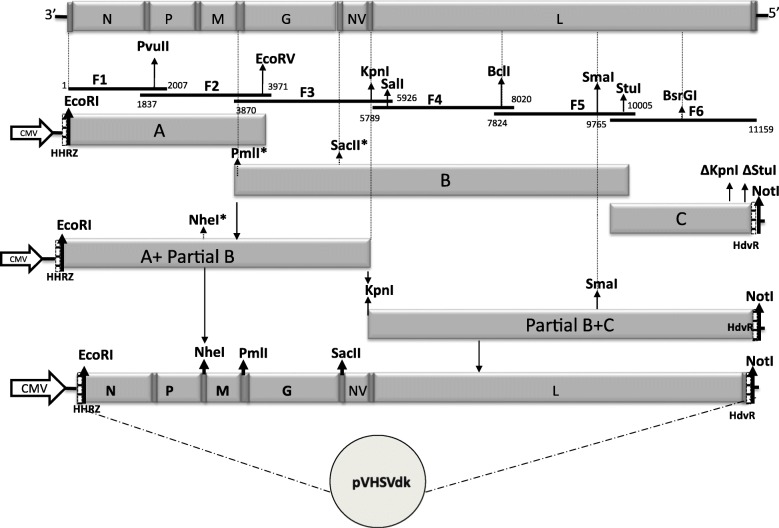
Construction of full-length cDNA clone of VHSV DK-3952B genome. Six overlapping cDNA fragments covering the entire VHSV genome were assembled by ligation into a pCI vector using the EcoRI, PvuII, EcoRV, KpnI, SalI, BclI, SmaI, StuI, BsrGI and NotI restriction sites. Assembly was carried out in 3 steps; (i) cloning of six fragments (F1-F6) separately in pGEM-T vectors (see methods), and addition of hammerhead ribozyme (HHRz) at the 5′-end of F1 and hepatitis delta ribozyme (HdvRz) at the 3′-end of F6; (ii) construction of A, B and C clones into the pCI vector; (iii) assembly of the full-length clone by ligating fragments from these three clones in the pCI vector. Restriction sites artificially created (*) and naturally present in the genome are indicated. Abbreviation: CMV, cytomegalovirus immediate-early enhancer and promoter

Synthesis and cloning of overlapping cDNA fragments from the RNA genome of VHSV strain DK-3592B was essentially carried out as described for the MI03 strain [7], except that some of the oligonucleotides primers used for RT-PCR amplification and mutagenesis were different. Table 1 shows the oligonucleotide primers used for cloning, sequencing and mutagenesis of various clones of strain DK-3592B and their location in the genome. The cDNA fragments obtained after RT-PCR amplification were cloned into pGEM-T or pGEM-T Easy vectors (Promega Corp., Madison, WI). From these subclones, a full-length cDNA copy of the DK-3592B RNA genome was constructed by assembling six overlapping cDNA fragments by standard cloning procedure using natural or artificially created unique restriction sites in the overlapping regions of the clones (Fig. 1).

**Table 1:** Oligonucleotides used for cloning, sequencing and mutagenesis of VHSV DK-3592B cDNA fragments

Primers	Sequences	Nucleotide no.^a^
VHSV-DK genome primers		
VHSV 1F	GTATCATAAAAGATGATGAGT	1–21
VHSV 1R	CAGCTTGAACTTCTTCATGGC	2007–1987
VHSV 2F	AAGAAGACCGACAACATACTCT	1837–1858
VHSV 2R	GACGAAACTTTGAGAGGAGAAA	3971–3950
VHSV 3F	ATCTCATCACCAACATGGCTCAAA	3870–3893
VHSV 3R	TTGTTCGCCTCTCCCCTAATTGT	5926–5888
VHSV 4F	CCGCTATTGACTTGCTCAAATTG	5789–5812
VHSV 4R	CTGATCCATGGTGGCTATGTGAT	8020–7998
VHSV 5F	AGATGATTGTCTCCACCATGAA	7824–7845
VHSV 5R	GAGATCCGCTCTCGTTCATCAA	10,005–9984
VHSV 6F	GACAAGAAAGCTGGGAAGAGA	9765–9785
VHSV 6R	GTATAGAAAATAATACATACCA	11,159–11,138
VHSV 1MF	GGACAAGATGATCAAGTACATC	595–616
VHSV 2MF	CCATTCTCTGCGAAGATCAACA	2435–2456
VHSV 3MF	GATAAAGAAACATGGCGACCCA	4547–4568
VSHV 4MF	GGATCATCAGGAAGAGACACCA	6392–6413
VHSV 5MF	AGGCACACCACTTCACCATCCA	8412–8433
VHSV-DK 5.3R	CCTTGGATTCTTGTTGAGTCA	5280–5260
Mutagenesis primers		
VHSV-DK PmlF	TTGTGTATACAACACGTGAGACCCACAATG	
VHSV-DK PmlR	CATTCCATTGTGGGTCTCACGTGTTGTATACACAA	
VHSV SacIIF	GTCTGAACACACCGCGGCGAATGACCACAATTCC	
VHSV SacIIR	GGAATTGTGGTCATTCGCCGCGGTGTGTTCAGA	
VHSV NheF	CCTCAGACAAGCTAGCACAAAAAAC	
VHSV NheR	GTTTTTTGTGCTAGCTTGTCTGAGG	

^a^The positions where the primers bind (nucleotide numbers) are according to the published sequence of VHSV DK-3592B genome in the GenBank under the accession number KC778774 (Yusuff and Vakharia, 2013)

Assembly of the full-length cDNA clone of VHSV DK-3592B into the pCI vector was carried out as described earlier [13]. The hammerhead ribozyme (HHRz) and hepatitis delta virus ribozyme (HdvRz) sequences were fused with the F1 and F6 fragments respectively by overlapping PCRs. The F1 fragment was digested with EcoRI and NotI and transferred into the pCI vector. The F2 fragment was ligated with F1 after digesting with PvuII and NotI restriction enzymes. The F2 and F3 fragments were ligated in vitro and then amplified with PmlIF and SacIIR primers. The amplified product was added to F1 and partial F2, which were already in pCI vector by inserting between the EcoRV and KpnI restriction sites. A small fragment from F3 was amplified with SacIIF and 5.3R primers and added to the above construct in pCI vector between SacII and KpnI sites to create “A + Partial B” clone. A portion of F2 was amplified with 2F + NheR and NheF +2R primer pairs by PCR (to create a NheI site) and this fragment was ligated after digesting with PvuII and EcoRV in the “A + partial B” clone. The “B” clone was generated by ligating F3, F4, and F5 fragments into pCI vector after digesting with indicated restriction enzymes (Fig. 1). To construct modified clone “C”, a portion of the F6 gene fragment from BsrGI to NotI (864-nt) was synthesized (Biomatik, Canada) in which the restriction sites KpnI and StuI present in the wild-type clone (at nucleotide positions 10,953 and 11,097, respectively) were deleted. DNA fragments of SmaI to StuI from F5 and StuI to BsrGI from F6 were amplified with specific primers to obtain a 1315-nt fragment. This fragment was digested with SmaI and BsrGI restriction enzymes and cloned into clone “C” between the same sites. Then, clone “B” was digested with KpnI and SmaI enzymes and the resulting fragment was cloned into the modified “C” clone to form “Partial B + C” clone. This clone was digested with KpnI and NotI restriction enzymes and this fragment was ligated to clone “A + Partial B” that was digested with the same enzymes. Finally, a full-length plasmid of VHSV DK-3592B strain was obtained and the DNA was completely sequenced for its integrity, using an automated DNA sequencer (Applied Biosystems, Foster City, CA, USA). This full-length plasmid was labeled as pVHSVdk (Fig. 1).

Unique restriction enzyme sites such as NheI, PmlI (compatible with PvuII) and SacII were created by substitutions of specific nucleotide(s) in the intergenic regions of the genome at positions 2233, 2945 and 4492, respectively, by PCR-directed mutagenesis to facilitate substitution(s) of VHSV genes between the two infectious full-length cDNA clones of VHSV.

### Construction of various chimeric cDNA clones of VHSV

**Fig. 2:**
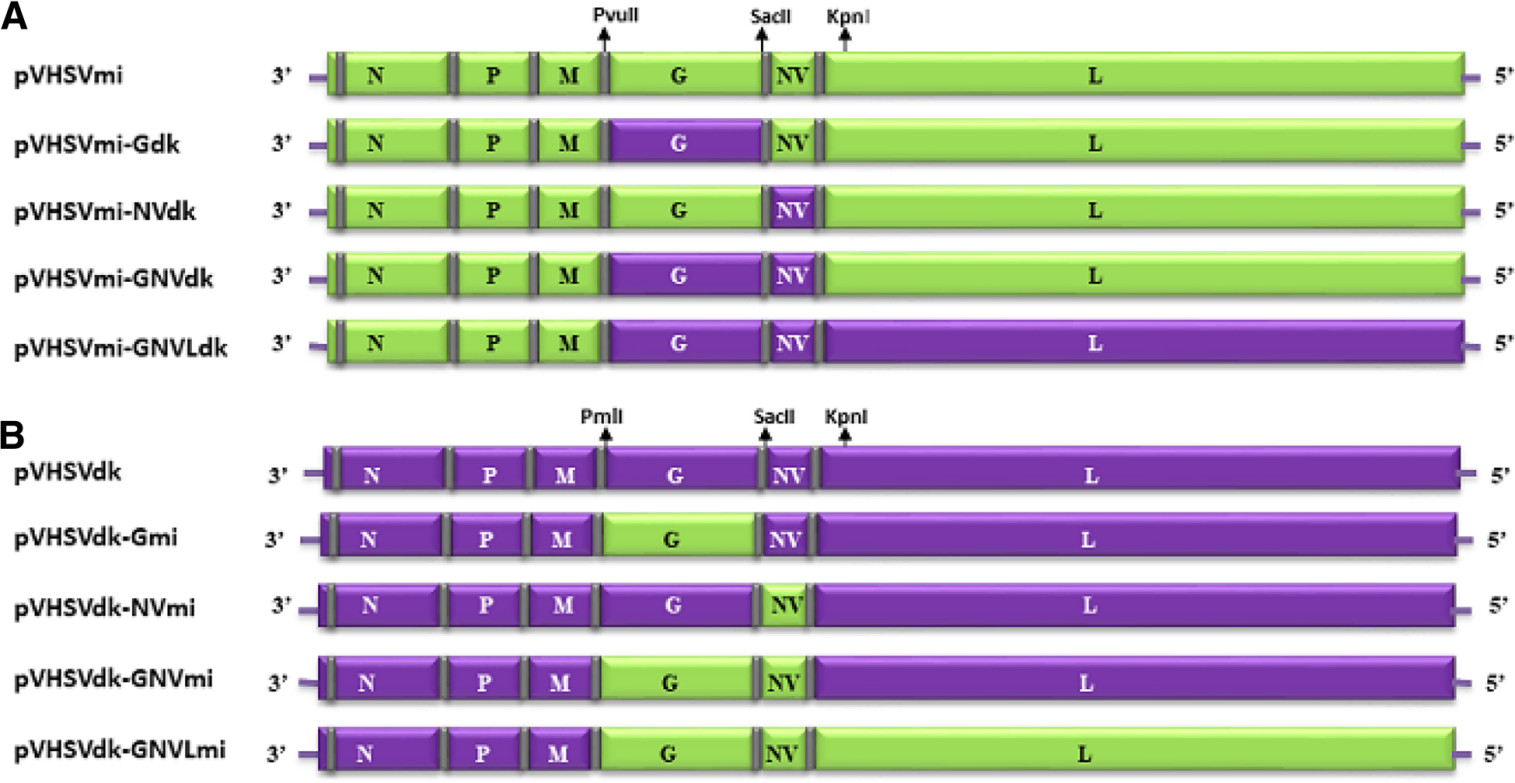
Schematic presentation of full-length cDNA constructs of VHSV strains MI03 (mi) and DK-3592B (dk) and various chimeric VHSVs. A map of the VHSV genome is shown at the top of panels A and B, depicting coding regions of N, P, M, G, NV and L genes that are separated by the flanking regulatory untranslated and intergenic sequences (vertical bars). Selected restriction sites, important for the construction of chimeric cDNA clones, are shown. These unique sites are present in the intergenic regions of the clones, which does not affect the coding regions of viral proteins. All these constructs contain a cytomegalovirus promoter at the 3′-end. A). Chimeric cDNA clones derived by substituting VHSV DK-3592B gene(s) into the pVHSVmi full-length clone. B). Chimeric cDNA clones derived by substituting VHSV-MI03 gene(s) into the pVHSVdk full-length clone. Light green boxes depict the coding regions of VHSV strain MI03, whereas, the purple boxes represent the coding regions of VHSV strain DK-3592B

Construction of a full-length infectious clone of VHSV-MI03 strain has been described previously [13]. This clone was modified and used as a backbone to introduce desired substitution(s) of the coding regions of different structural and non-structural genes of VHSV DK-3592B strain. Besides the unique NheI, PvuII and SacII restriction site present in the noncoding intergenic regions of the clone, a unique EcoRI site was destroyed by silent mutation in the L gene (at position 6164) of the full-length clone. This full-length plasmid of the VHSV MI03 strain was designated as pVHSVmi (Fig. 2a). To construct chimeric cDNA clones with pVHSVdk gene substitutions(s) into the pVHSVmi backbone, plasmid pVHSVmi was double-digested with restriction enzymes PvuII-SacII, or SacII-KpnI, and respective gene fragments of G and NV open reading frames (ORFs) were cloned between the same sites, respectively (Fig. 2). For ease of cloning, the NV gene was synthesized with intergenic sequences of the backbone clone containing the desired SacII-KpnI restriction sites. To insert the L gene, plasmid pVHSVmi was double-digested with KpnI and NotI (unique site in pCI vector) and the respective pVHSVdk L gene fragment was cloned into this plasmid.

Similarly, construction of chimeric cDNA clones with reciprocal pVHSVmi gene substitutions(s) into the pVHSVdk backbone plasmid was carried out as described above, except that PmlI site was used for cloning rather than PvuII (compatible with PmlI). DNA from all the above mentioned plasmids was sequenced by the dideoxy chain termination method to verify various gene substitution(s) in these clones.

### DNA transfection and recovery of chimeric viruses

To generate recombinant chimeric viruses, EPC cells were transfected with ten different plasmid constructs (see Fig. 2) along with the support plasmids using the protocol described earlier [13]. The same supporting plasmids, pN, pP, and pL of the MI03 strain, were used for transfection of both pVHSVmi and pVHSVdk derived clones. After transfection, the cells were washed and maintained in Eagle’s MEM containing 10% FBS at 14 °C for 5 days. Cell monolayers were observed for the development of virus-induced CPE. After 5 days of incubation, the cells were submitted to three cycles of freeze thawing. Supernatant was clarified by centrifugation at 8000×*g* in a microcentrifuge, and used to inoculate fresh cell monolayers in T-25 flasks at 14 °C. The supernatant was harvested and clarified for further characterization of the recombinant viruses.

### Preparation of virus stocks and plaque assay

To prepare recombinant virus stocks, confluent EPC cells grown in T-75 flasks at 25 °C were infected at an MOI of 0.01 in MEM with 5% FBS. After 1 h of adsorption at 14 °C, the inoculum was removed, and the cells were incubated at 14 °C until extensive CPE was observed. The supernatant was collected 4–5 days post-infection, clarified, and stored at − 80 °C. The titer of the virus was determined by plaque assay, as described [45]. The titer of the recombinant parental viruses (rVHSVmi and rVHSVdk) and chimeric viruses ranged from 1.2 X 10^6^ to 2.4 X 10^8^ plaque forming units (PFU)/ml.

### Confirmation of chimeric nature of the recombinant viruses

RT-PCR was performed on RNA extracted from the recovered viruses to confirm the chimeric nature of recombinant VHSVs in which the complete ORFs of the structural and non-structural genes were substituted between the two VHSV backbones whilst maintaining the intergenic regions of the individual full-length clones. Viral RNA was extracted from the supernatants of VHSV infected cell cultures, using an RNeasy® Mini Kit (Qiagen). RT-PCR was performed to verify the presence of specific region(s) of VHSV gene sequences substituted between the two clones, including artificially created restriction sites (NheI, PvuII, PmlI and SacII). The obtained RT-PCR products were directly submitted for DNA sequencing in the core facility of Institute of Marine & Environmental Technology to confirm the presence of the VHSV gene fragments and genetic markers introduced into the genome.

### Viral growth curves in cell culture

To analyze the in vitro growth characteristics of the recombinant VHSVs, confluent EPC cells were infected with the parental recombinant VHSVs of strains MI03 (rVHSVmi) and DK-3592B (rVHSVdk) or different chimeric recombinant VHSV stocks at an MOI of 0.01 in individual T-25 flasks. Virus present in the infected cell culture supernatant was collected at different time intervals, clarified by centrifugation, and titrated on EPC cells by plaque assay, as described earlier [45].

### Virus challenge experiments in juvenile rainbow trout

Two in vivo virus challenge experiments were conducted in compliance with guidelines provided by the Guide for the Care and Use of Laboratory Animals and the United States Public Health Service Policy on the Humane Care and Use of Laboratory Animals. Research-grade naive juvenile rainbow trout were provided by Clear Springs Foods, Inc., of Buhl, Idaho. Fish were reared to appropriate size in the Western Fisheries Research Center Aquatic Biosafety Level 2 wetlab on flow-through, sand-filtered, UV-irradiated lake water at 15 °C, and given pelleted feed (BioOregon) at 1.5% body weight per day. Feed was withheld for 24 h prior to the start of each experiment, and during this time fish were gradually acclimated to 12 °C. Fish were then transferred to the Aquatic Biosafety Level 3 laboratory of Western Fisheries Research Center, where all in vivo virus experiments were conducted following strict containment procedures.

Juvenile rainbow trout were challenged by intraperitoneal injection of virus in 25 or 50 μl of phosphate buffered saline (PBS). In experiment 1, fish of average weight 1 .3g were challenged in each of 11 treatment groups using a challenge dose of 1.5 × 10^5^ PFU per fish. In experiment 2, fish were 1 .6g average weight and there were 9 treatment groups challenged with a dose of 1.2 × 10^5^ PFU per fish. In each experiment, the positive control parental rVHSVmi and rVHSVdk, and all chimeric rVHSVs were tested in triplicate subgroups of 20 juvenile rainbow trout. The only exception to this was the repeat tests of rVHSVmi-Gdk and rVHSVdk-Gmi in experiment 2, which were done in duplicate groups of 15–18 fish due to limitations on fish numbers and tank space in the ABSL3 wetlab. Due to similar limitations, the positive control wild type parental virus strains wtVHSVmi and wtVHSVdk were tested as 1–3 tanks of 10–20 fish in the various experiments. As a negative control, each experiment included 2 or 3 tanks of 20 fish that were mock-infected by injection with PBS containing no virus. After injection, the individual replicate subgroups of fish were held in 30 L tanks with flow-through water at 12 °C, and fed three times per week. Fish were monitored daily for morbidity and mortality for a period of 30 days. Fish that died during the challenge were collected daily and disease signs were recorded in dead fish before saving them at − 80 °C. In each experiment a subset of fish that died in each treatment group (between 1 and 14 fish per treatment group, depending on the number of fish that died) were titered for infectious virus by plaque assay [45] to confirm the presence of virus at titers that suggested viral infection as the likely cause of death.

Differences in arc-sine transformed final cumulative percent mortality (CPM) values for all virus-challenged treatment groups were evaluated by ANOVA followed by a Tukey’s post-hoc test to define significant differences among treatment groups (implemented with PASW Statistics vV.18; IBM Analytics). Kinetics of survival of the fish in different groups within each experiment was analyzed using Kaplan-Meier estimation of survival curves, followed by comparison of the survival curves by log-rank test. *P* < 0.05 was considered significant for all tests. Mean day-to-death (MDD) was calculated for fish that died within each individual replicate tank, and then reported as the average MDD and standard deviation for replicate tanks within each treatment. For MDD calculations two fish in experiment 2 that died on day 1 post-challenge were excluded based on the likelihood that these fish died due to injury during injection rather than to virus infection.

### Determination of viral infection status at 7 days post-challenge

In rainbow trout challenge experiments 1 and 2, on day 7 post-challenge 3 fish were euthanized from each tank in the rVHSVmi series treatment groups. This resulted in sets of 9 fish for most treatment groups (or 6 fish for groups with duplicate tanks) that were stored at − 80 °C and then titered for infectious virus by plaque assay as described above. The viral titers determined in individual fish were compared with the amount of virus injected into the fish for each experiment to provide evidence for viral replication in vivo rather than just persistence of the inoculum virus. The detection limit of the plaque assay is 200 PFU per gram of fish.

### Determination of viral infection status in challenge survivors

At the end of experiment 1, on day 32 post-challenge, 9 surviving fish were euthanized from the rVHSVmi and wtVHSVmi treatment groups and stored frozen at − 80 °C. Similarly, at the end of experiment 2, on day 30 post-challenge, 9 surviving fish were euthanized from the rVHSVmi group and stored at − 80 °C. Also in experiment 2, the single surviving fish in the rVHSVdk treatment group was euthanized and stored. These fish were titered by plaque assay as above, to assess viral clearance relative to the prevalence of virus detected in these groups at 7 days post-challenge.

### Characterization of the chimeric viruses recovered from infected fish

For each treatment group that had mortality in these 2 experiments, viral RNA from one fish that died late in the monitoring period was sequenced to verify that recovered viruses contained the expected genome sequences. Genomic RNA was extracted from partially purified virus using an RNeasy Mini Kit (Qiagen), and subjected to RT-PCR amplification to obtain cDNA fragments of the VHSV genome, as described earlier. These DNA fragments were purified and sequenced to confirm the presence of introduced substitutions of different structural and non-structural protein genes into the VHSV genomes.

## Results

### Cloning and sequence analysis of VHSV strain DK-3592B

To prepare an infectious cDNA clone of the trout-virulent VHSV strain DK-3592B, overlapping cDNA fragments of genome were amplified, cloned and their DNA sequenced. We determined the complete nucleotide (nt) sequence of the DK-3592B strain and deposited it in GenBank under the accession no. KC778774. The trout-virulent DK-3592B genome (genotype Ia) is 11,159 nt in length. Comparison with the trout-avirulent MI03 strain genome (genotype IVb) revealed an overall difference of 13% at the nt level, with differences distributed throughout both coding and non-coding regions of all genes, as detailed in Table 2. The DK-3592B strain was shorter overall by 25 nt, due to deletions relative to MI03 at 5 locations that were all in non-coding regions. Comparison of the deduced amino acid sequences of the DK-3592B strain proteins with those of the MI03 strain showed 93% identity among the N, P and G proteins; > 96% among the M and L proteins; and 74% between the NV protein, which was the most divergent.

**Table 2:** Nucleotide and amino acid sequence differences between the complete genome sequences of VHSV strains MI03 and DK-3592B

Genomic region	# nt diffs/ total # nt, (%)	Deletions in DK-3592B relative to MI03	# aa diffs/ total # aa, (%)
3′ leader	2/53, (3.77%)		
N gene 5’UTR	6/111, (5.40%)		
N gene ORF	134/1212, (11.06%)		29/404, (7.18%)
N gene 3′ UTR	30/60, (50.0%)	20 nt	
N-P intergenic	0/2 (CG)		
P gene 5’UTR	7/57, (12.28%)	1 nt	
P gene ORF	98/666, (14.71%)		15/222, (6.76%)
P gene 3′ UTR	6/34, (17.65%)		
P-M intergenic	0/2 (CG)		
M gene 5’UTR	13/81, (16.05%)		
M gene ORF	68/603, (11.28%)		9/201, (4.48%)
M gene 3′ UTR	12/54, (22.22%)	1 nt	
M-G intergenic	0/2 (TG)		
G gene 5’UTR	7/33, (21.21%)		
G gene ORF	205/1521, (13.48%)		37/507, (7.30%)
G gene 3′ UTR	12/51, (23.53%)		
G-NV intergenic	0/2 (TG)		
NV gene 5’UTR	6/21, (28.57%)		
NV gene ORF	79/366, (21.58%)		32/122, (26.23%)
NV gene 3′ UTR	6/31, (19.35%)		
NV-L intergenic	0/2 (TG)		
L gene 5’UTR	11/94, (11.70%)		
L gene ORF	760/5952, (12.77%)		66/1984, (3.33%)
L gene 3′ UTR	9/38, (23.68%)	2 nt	
5′ trailer	23/116 (19.83%)	1 nt	
Full Genome	1489/11184 (13.31%)	25 nt total	

### Construction of full-length and chimeric cDNA clones of VHSV

Construction of a full-length infectious clone of the trout-avirulent VHSV MI03 strain has been described previously [13]. This plasmid was labeled as pVHSVmi. A full-length cDNA copy of the trout-virulent DK-3592B strain was constructed by assembling six overlapping cDNA fragments by standard cloning procedure, using natural or artificially created unique restriction sites in the overlapping regions of the clones. This full-length plasmid was designated as pVHSVdk. These two parental infectious clones were used to create 8 chimeric clones in which individual viral genes, or selected combinations of genes, were exchanged in a reciprocal manner between the two clones. In our strategy, complete ORFs of the structural and non-structural genes were substituted between the two VHSV backbones whilst maintaining the untranslated regions and intergenic regions of the parental full-length clone. Figure 2 shows a schematic presentation of various full-length cDNA clones of VHSV constructed by exchanging the individual G or NV genes, or the combination of G and NV, or G, NV and L genes between pVHSVmi and pVHSVdk.

### Recovery and characterization of recombinant VHSV

We transfected EPC cells with the ten different plasmid constructs (8 chimeras and the two parental VHSV strains) and recovered a series of 10 recombinant VHSVs. To verify the chimeric nature of each modified rVHSV, RNA was extracted from these viruses and used to obtain RT-PCR products. Sequence analysis of the RT-PCR products confirmed the chimeric nature of the rVHSVs as designed.

### Replication kinetics of chimeric viruses in vitro

**Fig. 3:**
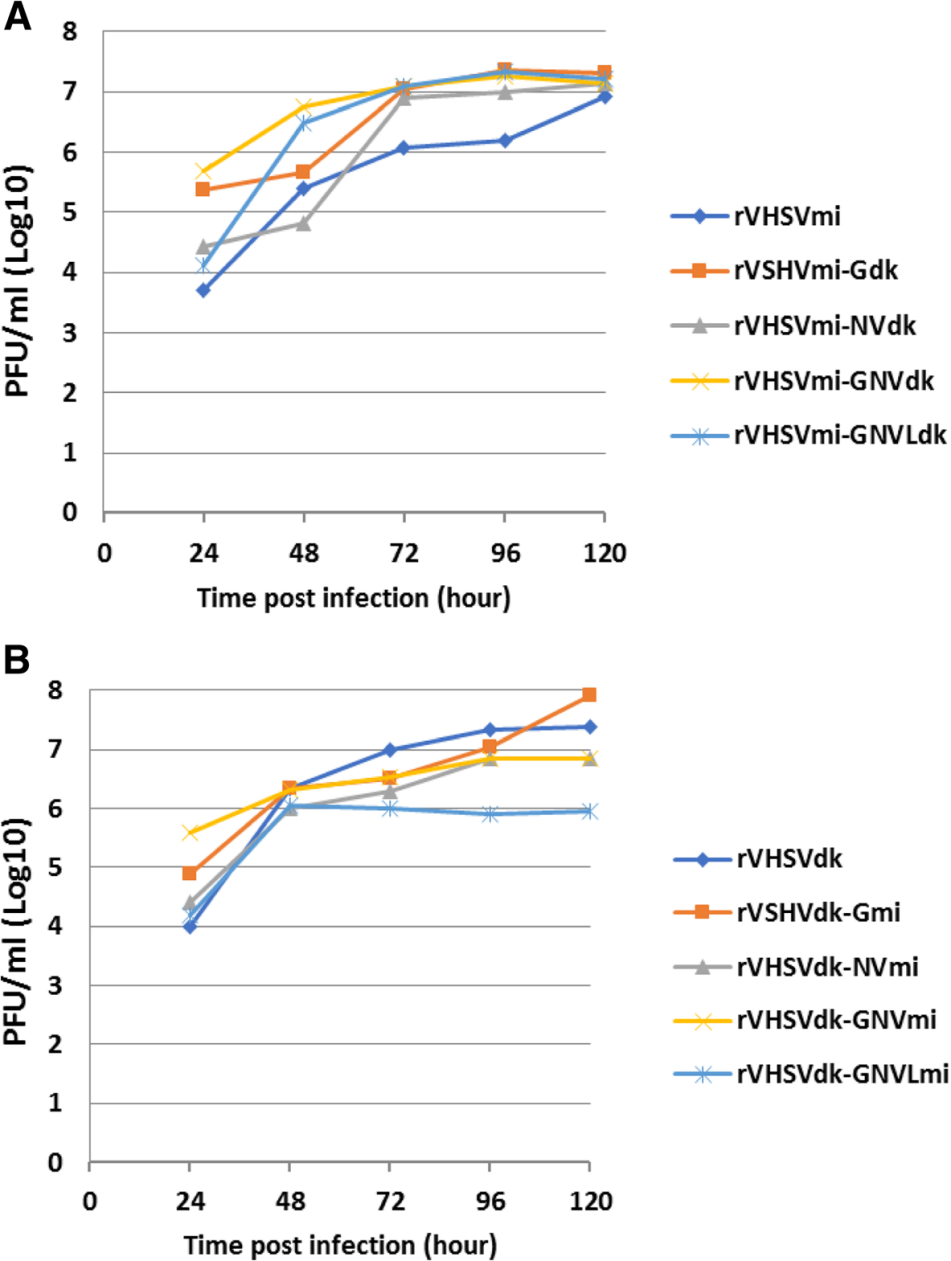
Replication kinetics of parental rVHSVmi, rVHSVdk, and chimeric VHSVs in vitro. Monolayers of EPC cells were infected at an MOI of 0.01 with the chimeric viruses harboring substitutions of specific VHSV gene(s) in the parental rVHSVmi (panel A) or rVHSVdk (panel B) viruses derived from respective pVHSVmi or pVHSVdk plasmids. The viruses were harvested at the indicated time points, and virus titers were determined by plaque assay

To analyze the in vitro growth characteristics of the parental rVHSVmi, rVHSVdk and their chimeric rVHSVs harboring substitutions(s) of specific VHSV gene(s), a one-step growth curve study was carried out in EPC cells. Figure 3 shows the growth curves of the parental rVHSVmi, rVHSVdk, and their derivatives at different time point 24–120 h post-infection (p.i). The results indicated that all rVHSVs were viable and replicated in vitro. Chimeric viruses of the rVHSVmi series grew to final titers very similar to the parental rVHSVmi (approximately 1 × 10^7.0^ PFU/ml) at 120 h p.i (Fig. 3 a). However, the parental rVHSVmi grew more slowly than the other rVHSVmi chimeric viruses, with titers approximately one log lower at 72 and 96 h p.i (Fig. 3 a). Titers of chimeric viruses of the rVHSVdk series were also generally similar to that of the parental rVHSVdk (approximately 1 × 10^7.5^ PFU/ml) at 120 h p.i, with the exception of rVHSVdk-GNVLmi, which replicated initially but then plateaued after 48 h and remained at a final titer approximately 1–2 logs lower than the other viruses (Fig. 3 b). Also the rVHSVdk-NVmi and rVHSVdk-GNVmi viruses grew slightly slower and had ~ 0.5 log lower final titer than rVHSVdk (Fig. 3 b). The chimeric virus rVHSVdk-Gmi had the highest final titer (1 × 10^8^ PFU/ml), but only at the final time point 120 h p.i, (Fig. 3 b).

### Virulence of recombinant VHSV in rainbow trout

**Fig. 4:**
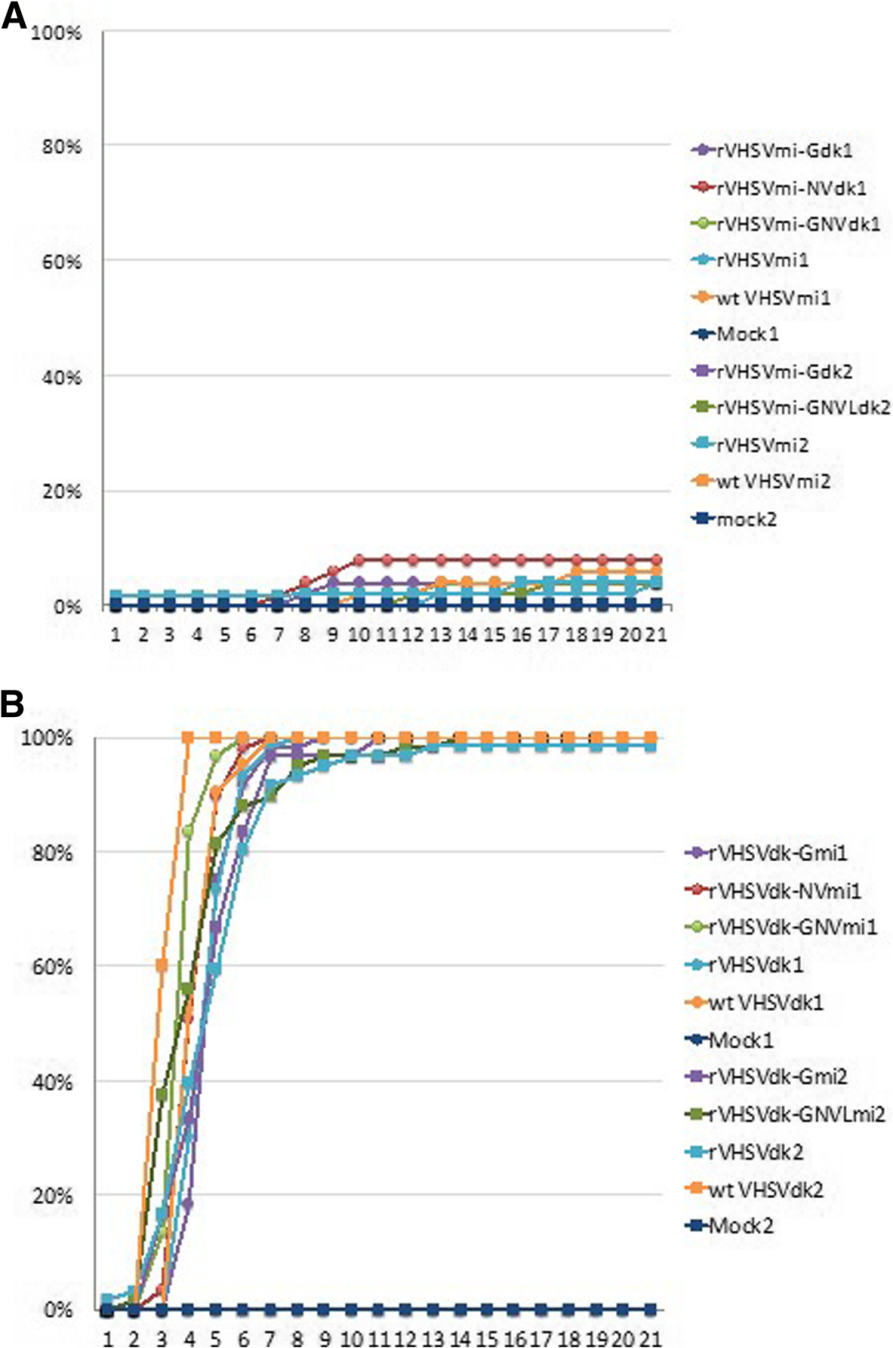
Virulence of parental rVHSVmi, rVHSVdk and chimeric rVHSVs in juvenile rainbow trout. Juvenile rainbow trout were challenged, as described in Table 3, by intraperitoneal injection with parental rVHSVmi or rVHSVdk, chimeric viruses with reciprocal gene exchanges, wild-type MI03 or DK-3592B viruses, or mock-challenged with PBS. Experiments 1 (circles) and 2 (squares) tested exchanges of the G, NV, G and NV, or G and NV and L genes. Mortality of fish after challenge was recorded daily and expressed as the average cumulative percent mortality (CPM) for triplicate subgroups of 20 fish. Variation among subgroups is provided in Table 3. A) rVHSVmi series groups for experiments 1 and 2, B) rVHSVdk series groups for experiments 1 and 2

The trout-virulence phenotype was tested in two experiments by intraperitoneal injection of reciprocal chimeric rVHSV into triplicate sub-groups of 20 juvenile rainbow trout for each treatment group, followed by daily monitoring for mortality and clinical signs of disease. The kinetics of mean cumulative percent mortality (CPM) in each treatment group are shown in Fig. 4, and final mortality and mean day-to-death values are shown in Table 3. In experiment 1, the trout-avirulent wild type MI03 strain (wtVHSVmi) and the corresponding parental recombinant virus rVHSVmi both caused extremely low mortality of 4–6% CPM as observed previously [13]. In the chimeric rVHSVmi treatment groups, replacement of the G, NV, or G and NV genes with the homologous genes from the virulent pVHSVdk clone did not affect the low virulence phenotype, resulting in 4–10% CPM (Fig. 4 a, Table 3). Similar results were obtained in experiment 2, where a repeat test of rVHSVmi-Gdk showed no mortality, and the chimeric virus rVHSVmi-GNVLdk, containing the G, NV, and L genes from the virulent pVHSVdk clone, caused an average of only 4% CPM (2 fish died in one of three replicate treatment tanks) (Fig. 4 a, Table 3).

**Table 3:** Summary results for in vivo challenge experiments

Treatment Group	Individual tank final CPM^a^	Ave. final CPM	Mean day to death±SD^b^	Day 7 infection, # pos/# tested (# > inoculum)^c^
Experiment 1				
wt VHSVmi	6, 0, 12	6	13.5*	8/9 (5)
rVHSVmi	0, 0, 12	4	19*	7/9 (3)
rVHSVmi-Gdk	12, 0, 6	6	14.5*	2/9 (0)
rVHSVmi-NVdk	0, 24, 6	10	11.6*	6/9 (3)
rVHSVmi-GNVdk	6, 0, 6	4	12.5*	4/9 (2)
wt VHSVdk	100	100	4.6*	nt
rVHSVdk	100, 100, 100	100	4.9 ± 0.20	nt
rVHSVdk-Gmi	100, 100, 100	100	5.2 ± 0.20	nt
rVHSVdk-NVmi	100, 100, 100	100	4.6 ± 0.28	nt
rVHSVdk-GNVmi	100, 100, 100	100	4.1 ± 0.15	nt
mock	0, 0	0	na	0/6
Experiment 2				
wt VHSVmi	0	0	na	nt
rVHSVmi	0, 6, 6	4	8.5*	3/9 (3)
rVHSVmi-Gdk	0, 0	0	na	2/6 (1)
rVHSVmi-GNVLdk	0, 12, 0	0	14.5*	3/9 (3)
wt VHSVdk	100	100	3.5*	nt
rVHSVdk	100, 100, 95	98	5.0 ± 0.34	nt
rVHSVdk-Gmi	100, 100	100	5.1*	nt
rVHSVdk-GNVLmi	100, 100, 100	100	4.64 ± 0.27	nt
mock	0, 0, 0	0	na	0/9

^a^Within each experiment, all treatment groups were tested as triplicate subgroups of 20 juvenile rainbow trout, with the exception of mock challenge negative control treatments or wild type strain positive control treatment that were tested in some experiments as single or duplicate groups (as indicated in column 2) due to space limitations in the aquatic Biosafety Level 3 wetlab. Also, duplicate subgroups of 20 fish were used for the reciprocal G exchange chimeras in experiment 2 because this was a repeat test of this treatment group from experiment 1

^b^Mean day-to-death (MDD) for individual tanks was used to calculate the group average MDD and standard deviation (SD). *Asterisks denote MDD for groups that had mortality data from only 1 or 2 tanks, either due to tanks with no mortality or less than 3 tanks in the treatment. In these cases, no SD could be calculated. “na” means all tanks in the treatment group had no mortality

^c^Infection status at 7 days post-challenge is shown for fish sampled from rVHSVmi-based viruses in experiments 1 and 2. For virus-positive fish, the number of fish in each group with viral loads greater than the challenge inoculum is shown in parentheses as an indication of the ability of the virus to replicate in vivo. The infectious viral loads determined by plaque assay are shown in Fig. 5

In reciprocal treatment groups based on the pVHSVdk clone, the results were also clear (Fig. 4 b). In experiment 1, the fish in positive control groups challenged with the wild type DK-3592B strain (wtVHSVdk) or the parental rVHSVdk had extremely rapid mortality beginning on day 3 and reaching 100% CPM by day 7. This confirmed that the rVHSVdk generated from the new infectious clone pVHSVdk was highly virulent in rainbow trout, as expected. Similar rapid mortality reaching 100% CPM was observed for chimeric rVHSVdk containing the G, NV, or G and NV genes from the trout-avirulent pVHSVmi clone. In experiment 2, the positive control groups had 98–100% CPM and the chimeric rVHSVdk-Gmi containing the G, or the rVHSVdk-GNVLmi, containing the G and NV and L genes from pVHSVmi had 100% CPM, again indicating no change in phenotype relative to the parental rVHSVdk.

Statistical analyses of final CPM data resolved the results from each experiment into two clearly distinct statistical groupings comprised of the VHSVmi series treatment groups (i.e. wtVHSVmi, rVHSVmi, and chimeric viruses based on exchange of genes from VHSVdk into the rVHSVmi clone) or the VHSVdk series groups (i.e. wtVHSVdk, rVHSVdk, and chimeric viruses based on exchange of genes from VHSVmi into the rVHSVdk clone), without exception. Analyses of survival kinetics generated similar results with the exceptions that the rVHSVdk-NVmi and rVHSVdk-GNVmi groups had faster mortality than rVHSVdk in experiment 1.

In experiments 1 and 2, the small numbers of fish that died in the rVHSVmi series treatment groups had mean day-to-death (MDD) values ranging from 8.5–19 days post-challenge (Table 3). In contrast, fish that died in the rVHSVdk series groups died more rapidly, with MDD of 4.1–5.1 days post-challenge. Titering confirmed virus as the likely cause of mortality in both experiments, with viral loads ranging from 4 × 10^2^ to > 4 × 10^7^ PFU/g of fish in the nearly all fish that died. The identity of the virus in each experimental group was confirmed by sequencing viral RNA from a fish that died late in the observation period for each group. There was no mortality in any mock-challenged control group in either experiment.

### Infection status of rainbow trout 7 days after challenge with low virulence rVHSVmi series viruses

**Fig. 5:**
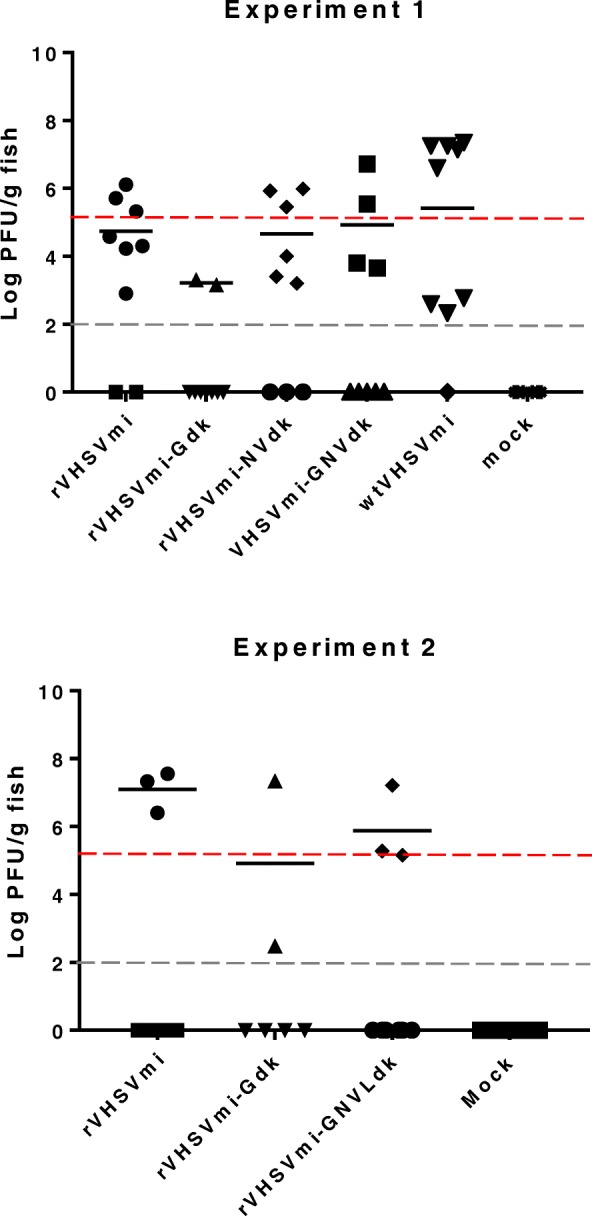
Infectious virus titers in rainbow trout 7 days after challenge with low virulence rVHSVmi series viruses in experiments 1 and 2. Juvenile rainbow trout in low virulence rVHSVmi series groups in experiments 1 and 2 were sampled at 7 days post-challenge and tested for infection status by plaque assay. Nine fish were sampled for each experimental group, except the mock group in experiment 1 and the rVHSVmi-Gdk group in experiment 2, each of which had 6 fish. Titers of infectious virus are presented for individual fish in each group with a horizontal bar indicating the mean of virus-positive fish. Red dotted lines indicate the amount of virus in the inocula injected into each fish, so that virus titers above this line indicate ability of the virus to replicate in vivo. The detection limit for the plaque assay is 100 PFU, as indicated by the gray dotted line

In experiments 1 and 2, fish were sampled from each tank in the rVHSVmi series groups on day 7 post-challenge, to assess ability of these low virulence viruses to persist and replicate in rainbow trout. This was not done for the rVHSVdk series groups because high mortality had already occurred by day 7, and titering of virus in fish that died confirmed ability of those viruses to replicate in vivo. For the rVHSVmi series, the proportion of fish that were virus-positive among those sampled on day 7 is shown in Table 3, and the infectious virus titers determined by plaque assay in individual fish are shown in Fig. 5. Virus was recovered from some fish in each treatment group, indicating that wtVHSVmi and all rVHSVmi recombinants in experiments 1 and 2 (rVHSVmi, rVHSVmi-Gdk, rVHSVmi-NVdk, rVHSVmi-GNVdk,and rVHVmi-GNVLdk) were able to persist in some fish for at least 7 days, despite causing very low mortality of less than 10%. Prevalence ranged from 2/9 to 8/9 fish positive for virus at day 7, and average virus titers varied from approximately 10^2^ to 10^7^ PFU/g. For each treatment group tested, there was at least 1 fish (between 1 and 5 fish per treatment group) with a titer higher than the original inoculum, indicating ability to replicate in vivo (note that for rVHSmi-Gdk, this was one fish in experiment 2). This indicates that wtVHSVmi and all rVHSVmi chimeras tested in experiments 1 and 2 were viable in vivo and replicated in at least some fish. No virus was detected in mock-challenged fish sampled at day 7.

### Clearance of the low virulence wtVHSVmi and rVHSVmi in challenge survivors

There was no virus detected by plaque assay in 9 surviving fish sampled from the rVHSVmi treatment groups at 30–32 days post-challenge in each of experiments 1 and 2. Similarly, there was no virus detected in 9 surviving fish sampled at 32 days post-challenge from the wtVHSVmi group in experiment 1. Compared with the positive detections of virus in these treatment groups at 7 days post-challenge (ranging 3/9 to 8/9, Table 3), this lack of detectable virus demonstrates clearance of the avirulent wtVHSVmi and rVHSVmi between 7 and 30–32 days post-challenge. Interestingly, we also tested the sole survivor of the high virulence rVHSVdk group in experiment 2 (average CPM 98%) and found no virus, suggesting that the single fish that was able to avoid mortality also cleared this highly virulent strain.

## Discussion

Reverse genetics studies have demonstrated that fish novirhabdovirus genomes, like mammalian rhabdovirus genomes, are extremely flexible at the molecular level, remaining viable despite various substitutions of homologous genes from other viruses, as well as insertions of foreign genes or NV gene deletion (reviewed in 41). Among novirhabdoviruses, reverse genetics systems have now been developed for three strains of VHSV [12, 13, 46], three strains of IHNV [47–49], and one strain of snakehead rhabdovirus (SHRV) [50]. The first aspect tested with many of these infectious clones was deletion of the NV gene. For two rIHNV systems and three rVHSV systems, NV deletion mutants have been created and shown to be viable but with reduced replication efficiency in fish cell lines, and attenuated virulence in relevant fish host species [11–13, 46, 47, 49].

Several chimeric recombinant viruses have been produced from modified novirhabdovirus clones in which genes were removed and replaced with the heterologous gene from another virus strain or species, often with low levels of nucleotide identity. This was first done with an infectious clone of a French IHNV strain 32/87 that has high virulence in rainbow trout [11, 47]. Using this IHNV clone, inter-species chimeras were created in which the G, NV, and M genes were independently substituted with the corresponding gene from a European strain of VHSV that also had high virulence in rainbow trout (genotype Ia). In each case, these chimeras were viable in vitro in fish cell lines, and they exhibited high virulence in vivo for rainbow trout [11, 40, 51]. In this same rIHNV system, other chimeras were produced containing the G genes from the fish rhabdovirus spring viremia of carp virus (SVCV), the mammalian rhabdovirus vesicular stomatitis virus (VSV), or various strains of VHSV or IHNV. In each case, viable chimeric recombinant viruses were recovered and shown to replicate in fish cell lines [41, 42, 51]. For those tested in vivo (all but the rIHNV-Gvsv), these chimeric rIHNVs had high virulence in rainbow trout [41–43]. In a different system, an infectious clone of snakehead rhabdovirus was used to produce a chimera with the G gene from IHNV, and the resulting chimeric rSHRV-Gihnv was viable in vitro [52]. To date, there are no reports of chimeric exchanges involving the large viral L protein, which comprises approximately half of the length of the viral genome. Thus our chimeras, in which the G, NV, and L genes were exchanged together, expand the known flexibility of the fish rhabdovirus genome to include as much as 70% of the viral genome, and 3 of the 6 genes.

These previous studies of chimeric recombinant viruses confirm the tolerance of fish rhabdovirus genomes to major genetic modifications. However, in many cases they do not provide information about virulence determinants because the parental viruses that contributed to the chimera did not differ in phenotype, but both had high virulence in rainbow trout. However, there have been four chimeric virus studies that generated data relevant to the role of the G and/or NV genes in trout-virulence. First, although the rIHNV-Gvhsv chimera was comprised of parental IHNV and VHSV strains that both had high trout-virulence, histological examination of rainbow trout that succumbed to infection with this chimeric virus revealed lesions that were reported as being more similar to those caused by IHNV rather than VHSV [40]. Another inter-species chimera, the rIHNV-Gsvcv, is especially informative for host-specific virulence because the parental viruses differ in phenotypes. The parental rIHNV clone has high virulence in trout and low virulence in carp and koi (both *Cyprinid carpio*), while the SVCV that provided the G gene has low virulence in trout and high virulence in carp and koi. In vivo testing found that rIHNV-Gsvcv was still highly virulent in trout, and it was not virulent in carp or koi, despite having the G gene of SVCV [41, 43]. This led these authors to suggest that something other than the viral G protein determined host-specific virulence. As another example, two chimeric rIHNV viruses carrying G genes from either trout-virulent or trout avirulent VHSV strains (genotypes Ia or Ib) did not differ when tested for virulence in rainbow trout [42]. In the same report, other chimeric rIHNV carrying the NV genes of the same two differential VHSV strains also showed no difference of virulence in trout. Finally, in vivo testing of intra-species rIHNV chimeras containing the G, NV, or G and NV genes from two North American IHNV strains with strong host-specificity for rainbow trout (M genogroup) or sockeye salmon (U genogroup) also indicated that host-specificity was not determined by the G or NV genes [41]. Collectively, these examples all suggest that the genetic basis of trout-virulence in fish novirhabdoviruses is not determined by the G or NV genes. This is supported by the results of our intra-species chimeric rVHSV as presented here.

In our strategy, we systematically exchanged the viral G, NV, and L genes, either individually or as selected gene combinations, between the trout-avirulent pVHSVmi clone (representing VHSV strain MI03, genotype IVb) and the trout-virulent pVHSVdk clone (representing VHSV strain DK-3592B, genotype Ia). All recombinant viruses were viable and replicated in cell culture. In challenge experiments, chimeric rVHSVs with reciprocal exchanges of the G, NV, G and NV, or G and NV and L genes together, all demonstrated ability to infect and replicate in rainbow trout, albeit with varying efficiencies. However, none of the chimeras showed any change of the virulence phenotypes for the contrasting parental clones in juvenile rainbow trout. We therefore expand on the previously published observations to suggest that the G, NV and L genes do not determine trout-virulence for VHSV. With regard to the report of a virulence determinant within the VHSV L protein (I1012F) that changes in vitro virulence [53], our two VHSV strains did differ at this site, with a Phe residue in rVHSVdk and an Ile residue in rVHSVmi. However, when we exchanged the L genes along with the G and NV genes, the chimeric rVHSVdk-GNVLmi did not exhibit any loss of trout-virulence, and rVHSVmi-GNVLdk did not show any gain of virulence. Thus, this residue was not a determining factor for in vivo trout-virulence as tested here.

We show here that the open reading frames of the G, NV, and L genes, in combination, do not determine trout-virulence of VHSV, suggesting 70% of the viral genome is not involved with this phenotype. This means that trout-virulence determinants must lie elsewhere, potentially in the N, P, or M genes at the 3′ end of the viral genome. It is also possible that host-specific virulence determinants are based on non-coding regions, such as the genome leader, trailer, or untranslated regions in the viral genes. In addition, it could be that virulence determinants do exist in the G, NV, and/or L gene(s), but require interaction with additional determinants that lie elsewhere.

In the past, it has often been assumed that the viral G gene is an important determinant of host-specificity and virulence of VHSV and other novirhabdoviruses because it encodes the single viral surface glycoprotein that interacts with the host cell receptor. Receptor recognition mechanisms of host specificity and virulence have been demonstrated for other fish viral pathogens, including infectious pancreatic necrosis virus (IPNV) and betanodaviruses. For IPNV, virulence determinants have been identified as specific amino acids in the viral VP2 protein that appear to fold at surface locations on the viral coat protein [54, 55]. For betanodaviruses, reverse genetics systems have generated reassortants that conclusively show host specificity is determined by a C-terminal region in the viral coat protein gene that is also predicted to protrude from the protein surface [56–58]. The idea that novirhabdovirus host-specific virulence does not involve the G gene suggests a very different mechanism that does not involve the viral coat protein recognition of host cell receptors. Elucidation of that mechanism will be a major goal for future work.

## Conclusions

We cloned and sequenced the VHSV genome of the DK-3592B strain and prepared an infectious clone of the trout-virulent strain. To identify the molecular basis of VHSV virulence in rainbow trout, we utilized an existing infectious clone of the trout-avirulent MI03 strain and constructed eight chimeric VHSV clones in which the coding region(s) of glycoprotein (G), non-virion protein (NV), G and NV, and G, NV and polymerase (L) genes were exchanged between the two clones. Using the reverse genetics approach, we generated ten recombinant VHSVs from cloned cDNA constructs, and characterized the recovered rVHSVs in vitro and in vivo. Our results indicate that G, NV and L genes of VHSV are not, by themselves or in combination, major determinants of host-specific virulence in trout. Hence, additional studies are warranted to identify the function of VHSV N, P, and M protein(s), or non-coding regions, in trout-virulence.

## Abbreviations

ANOVA: Analysis of Variance
cDNA: Complementary DNA
CMV: Cytomegalovirus
CPE: Cytopathic effect
CPM: Cumulative percent mortality
cRNA: Complementary RNA
EPC: *Epithelioma papulosum cyprini*
FBS: Fetal bovine serum
G: Glycoprotein
HdvRz: Hepatitis delta virus ribozyme
HHRz: Hammerhead ribozyme
IHNV: Infectious hematopoietic necrosis virus
IPNV: Infectious pancreatic necrosis virus
L: Large protein or polymerase
M: Matrix protein
MDD: Mean day-to-death
MEM: Minimal essential medium
MOI: Multiplicity of infection
N: Nucleoprotein
NV: Non-virion protein
ORF: Open reading frame
P: Phosphoprotein
p.i.: Post-infection
PASW: Predictive Analytics SoftWare
PBS: Phosphate buffered saline
PCR: Polymerase chain reaction
PFU: Plaque forming unit
RT-PCR: Reverse transcription polymerase chain reaction
SHRV: Snakehead rhabdovirus
SVCV: Spring viremia carp virus
VHSV: Viral hemorrhagic septicemia virus
VSV: Vesicular stomatitis virus
